# Analysis of factors influencing diabetic peripheral neuropathy in patients with type 2 diabetes mellitus and construction of a nomogram prediction model

**DOI:** 10.3389/fneur.2026.1842867

**Published:** 2026-06-24

**Authors:** Shangren Xie, Jieru Wu, Yaoqi Xu, Feifei Lin, Rui Huang, Yingsong Zheng

**Affiliations:** 1 Department of Traditional Chinese Medicine, Wenzhou Central Hospital, Wenzhou, Zhejiang, China; 2 Department of Acupuncture and Tuina, Wenzhou Central Hospital, Wenzhou, Zhejiang, China; 3 Department of ENT Head and Neck Surgery, Wenzhou Central Hospital, Wenzhou, Zhejiang, China

**Keywords:** diabetic kidney disease, influencing factors, nomogram, peripheral neuropathy, type 2 diabetes mellitus

## Abstract

**Objective:**

To analyze the influencing factors of diabetic peripheral neuropathy (DPN) in patients with type 2 diabetes mellitus (T2DM) and to construct a nomogram.

**Methods:**

A total of 570 patients with T2DM were included and assigned to a training set (285 cases) and a validation set (285 cases) in a 1:1 ratio. Logistic regression analysis was used to identify independent risk factors, and a nomogram was developed, validated, and evaluated.

**Results:**

The advanced age (OR = 4.575), body mass index ≥ 24.0 kg/m^2^ (OR = 2.463), history of smoking (OR = 2.585), history of drinking (OR = 2.717), concomitant hypertension (OR = 2.925), concomitant dyslipidemia (OR = 2.393), concomitant DKD (OR = 2.602), and concomitant DR (OR = 4.030) were independent risk factors for DPN in T2DM (*p* < 0.05). In the training set and validation set, the area under the ROC curve was 0.795 (95% *CI*: 0.740–0.850) and 0.857 (95% *CI*: 0.812–0.903), respectively. Both calibration curve and actual curve conformed to the ideal curve. Hosmer-Lemeshow goodness of fit test showed χ^2^ = 8.212 and 8.392, respectively, *p* = 0.413, 0.396, respectively. When the threshold probabilities were between 0.22–0.96 and 0.18–1.00, the nomogram was more favorable for predicting the risk of DPN in T2DM.

**Conclusion:**

Advanced age, body mass index ≥24.0 kg/m^2^, history of smoking, history of drinking, comorbid hypertension, comorbid dyslipidemia, comorbid DKD, and comorbid DR are independent risk factors for DPN in T2DM. The nomogram based on these factors can effectively predict the risk of DPN in patients with T2DM.

## 1 Introduction

Type 2 diabetes mellitus (T2DM) is a major global public health issue, imposing a heavy burden on individuals with T2DM, their families, and society (1, 2). T2DM can be complicated by diabetic peripheral neuropathy (DPN) (3). DPN is chronic and insidious, and it progressively worsens (4). Patients with T2DM often lack specific clinical symptoms in the early stages of DPN, typically presenting with numbness, pain, and other symptoms, by which time the condition may already be irreversible (5). Furthermore, the pathogenesis of DPN is complex, and pattern differentiation and treatment based on Traditional Chinese Medicine (TCM) principles are key to effectively managing diabetic peripheral neuropathy (DPN). Among the various TCM syndrome types, the pattern of qi deficiency and blood stasis is relatively common. It is characterized by symptoms such as fatigue, physical weakness, obesity, shortness of breath with reluctance to speak, a pale tongue coating, and a thready, choppy pulse. This condition can seriously endanger patients’ health and quality of life; however, effective treatment strategies are still lacking. Early detection, diagnosis, and treatment of DPN can help prevent adverse outcomes such as disability and amputation. Therefore, there is an urgent need to identify independent risk factors for DPN in T2DM patients and to explore effective tools for early screening of DPN. Nomograms can provide individualized risk assessments, and studies have confirmed that nomograms can be used for individualized prediction of frailty risk in diabetic patients (6), peritoneal dialysis-related peritonitis (7), and osteoporosis in T2DM patients (8). Based on this, the present study aims to analyze the influencing factors of DPN in T2DM patients and construct a nomogram to predict the risk of DPN in T2DM patients, with the goal of providing an efficient tool for early and accurate identification of T2DM patients at high risk of DPN.

## 2 Materials and methods

### 2.1 Research subjects

The sample size was estimated according to equation $n=\frac{{Z_{\alpha /2}}^{2}(1-P)P}{\delta^{2}}$ , where *α* represents the two-sided significance level and was set at 0.05. The value of *Z*_*α*/2_ (i.e., *Z*_0.05/2_) was obtained from the standard normal distribution table as 1.960. *P* represents the estimated target population proportion. Based on the literature (3) and the preliminary experiment, the proportion of T2DM patients complicated with DPN was estimated to be approximately 65%. *δ* represents the allowable error, which was set as 0.1 times *P* in this study, namely 0.065. Substituting these values into the above formula yielded *n* ≈ 207. Note: the sample size may be increased according to the actual situation.

A total of 570 patients with T2DM admitted to our hospital from June 2022 to June 2024 were selected. These 570 T2DM patients were randomly divided into a training set (285 cases, exceeding the minimum sample size of 207 cases) and a validation set (285 cases, exceeding the minimum sample size of 207 cases) in a 1:1 ratio.

Inclusion criteria: ① Diagnosis of T2DM according to the diagnostic criteria (9); ② Age ≥18 years; ③ The Traditional Chinese Medicine (TCM) syndrome is classified as qi deficiency with blood stasis; ④ Clear consciousness and the ability to communicate normally.

**Figure 1:**
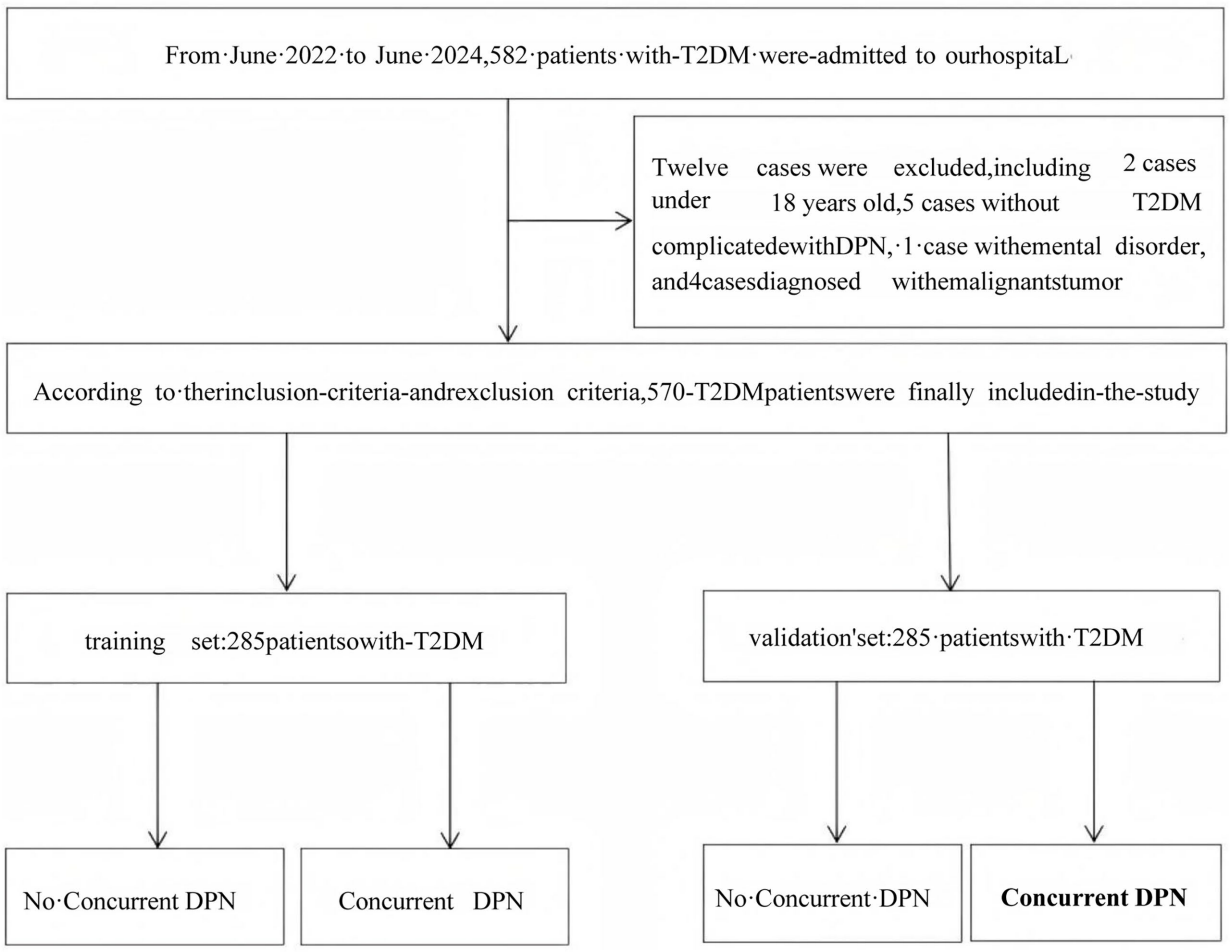
Flow chart for inclusion of study subjects.

Exclusion criteria: ① DPN caused by other factors, such as cerebral infarction; ② Malignant tumors; ③ Mental disorders (see Figure 1).

This study was approved by the hospital’s ethics committee.

### 2.2 Research methods

1. Data collection: Gender, age, body mass index (BMI), marital status, household registration type, education level, family monthly per capita income, smoking history, alcohol consumption history, comorbidity of hypertension, comorbidity of dyslipidemia, comorbidity of diabetic kidney disease (DKD), comorbidity of diabetic retinopathy (DR), medication regimen, systolic blood pressure (on the day after admission), diastolic blood pressure (on the day after admission), fasting blood glucose (on the day after admission), glycated hemoglobin (on the day after admission), creatinine (on the day after admission), total cholesterol (on the day after admission), and triglycerides (on the day after admission).
2. Differential diagnostic methods for DPN (All included patients with T2DM underwent a detailed neurological evaluation by the same trained neurologist). (1) Detailed medical history collection was performed to exclude patients with a history of familial neuropathy, alcoholic neuropathy (daily alcohol intake >50 g of pure ethanol for >2 years), vitamin B12 deficiency (serum vitamin B12 < 200 pg./mL), hypothyroidism (TSH > 4.2 mIU/L), uremia (serum creatinine >707 μmol/L), or exposure to drugs or toxins, such as chemotherapeutic agents or heavy metals. (2) Neurological physical examination was performed, and the Michigan Neuropathy Screening Instrument was used for preliminary screening. Patients with positive screening results further underwent nerve conduction velocity testing. Patients with non-length-dependent, asymmetric, or acute-onset neuropathy were excluded from the diagnosis of DPN. (3) Neuroelectrophysiological examination was performed. Electromyography and nerve conduction velocity testing were conducted in all patients clinically diagnosed with DPN. Patients were excluded if they presented with any of the following features inconsistent with typical DPN: ① markedly slowed motor nerve conduction velocity (<60% of the lower limit of normal); ② conduction block or waveform dispersion; and ③ asymmetric or multifocal distribution. (4) Imaging examination was performed. For patients suspected of having neurological symptoms caused by cerebral infarction, lumbar disc herniation, cervical spondylosis, or other conditions, cranial or spinal MRI was performed to clarify the etiology. (5) Laboratory examination was performed. Serum vitamin B12, folic acid, thyroid function, immunoglobulins, antinuclear antibodies, antineutrophil cytoplasmic antibodies, and serum and urine immunofixation electrophoresis were routinely tested to exclude immune-mediated, nutritional deficiency-related, or paraproteinemia-associated neuropathy. After the above differential diagnostic procedures, all cases of peripheral neuropathy caused by non-diabetic etiologies were excluded.
3. Grouping basis (9, 10): Investigate the occurrence of DPN in T2DM patients in the training and validation sets, and further divide them into DPN group and non-DPN group.
4. Quality control: (1) All group members participating in the survey were trained uniformly, and those who passed the assessment at the end of the training were included. (2) Data entry was performed by two personnel using double entry. (3) After data entry, 20% of the data was randomly selected for accuracy and completeness checks.

### 2.3 Statistical methods

Statistical analysis was performed using SPSS 25.0, and a *p*-value of <0.05 was considered statistically significant. Categorical data were described using frequency (%), and chi-square tests were used. For normally distributed continuous data, means ± standard deviations were used, and an independent-samples *t*-test was conducted. Logistic regression analysis was performed using the occurrence of DPN in 285 T2DM patients in the training set as the outcome variable to identify independent risk factors for DPN in T2DM patients. The identified independent risk factors were entered into R3.6.3 software using the rms package to construct a nomogram for predicting DPN risk in T2DM patients. The nomogram constructed in this study was validated using the data from the validation set. ROC curves were plotted to evaluate discrimination, calibration was assessed using the calibration curve and Hosmer-Lemeshow goodness-of-fit test, and decision curve analysis (DCA) was used to evaluate clinical net benefit.

## 3 Results

### 3.1 T2DM patients with DPN

Among the 285 T2DM patients in the training set, 186 (65.26%) had DPN, and 99 (34.74%) did not.

Among the 285 T2DM patients in the validation set, 194 (68.07%) had DPN, and 91 (31.93%) did not.

### 3.2 Analysis of general data of DPN and non-DPN groups in the training set, and general data of training and validation sets

There were no statistically significant differences between the DPN group and non-DPN group in the training set with regard to gender, marital status, household registration type, education level, family per capita monthly income, medication regimen, diastolic blood pressure, fasting blood glucose, and glycated hemoglobin (*p* > 0.05). However, significant differences were found in age, body mass index, smoking history, drinking history, comorbidity of hypertension, comorbidity of dyslipidemia, comorbidity of diabetic kidney disease (DKD), comorbidity of diabetic retinopathy (DR), systolic blood pressure, creatinine, total cholesterol, and triglycerides (*p* < 0.05). No statistically significant differences were found in gender, age, body mass index, marital status, household registration type, education level, family per capita monthly income, smoking history, drinking history, comorbidity of hypertension, comorbidity of dyslipidemia, comorbidity of DKD, comorbidity of DR, medication regimen, systolic blood pressure, diastolic blood pressure, fasting blood glucose, glycated hemoglobin, creatinine, total cholesterol, and triglycerides between the training set and the validation set (*p* > 0.05). These results are shown in Table 1.

**Table 1:** General data analysis of DPN and non-DPN groups in the training set, and general data of training and validation sets (cases (%)/mean ± SD).

Factors	Concurrent DPN group (*n* = 186)	No concurrent DPN group (*n* = 99)	*χ* ^2^ */t*	*P*	Training set (*n* = 285)	Validation set (*n* = 285)	*χ* ^2^ */t*	*P*
Gender			2.786	0.095			0.948	0.330
Male	113 (60.75)	70 (70.71)			183 (64.21)	194 (68.07)		
Female	73 (39.25)	29 (29.29)			102 (35.79)	91 (31.93)		
Advanced age			7.343	0.007			0.473	0.492
Yes	24 (12.90)	3 (3.03)			27 (9.47)	32 (11.23)		
No	162 (87.10)	96 (96.97)			258 (90.53)	253 (88.77)		
Body mass index			8.479	0.004			0.691	0.406
≥24.0 kg/m^2^	140 (75.27)	58 (58.59)			198 (69.47)	207 (72.63)		
<24.0 kg/m^2^	46 (24.73)	41 (41.41)			87 (30.53)	78 (27.37)		
Marital status			2.362	0.501			0.675	0.879
Unmarried	4 (2.15)	3 (3.03)			7 (2.46)	9 (3.16)		
Married	171 (91.94)	86 (86.87)			257 (90.18)	251 (88.07)		
Divorce or separation	4 (2.15)	5 (5.05)			9 (3.16)	11 (3.86)		
Widowed	7 (3.76)	5 (5.05)			12 (4.21)	14 (4.91)		
Type of household registration			0.234	0.628			0.269	0.604
Agricultural	66 (35.48)	38 (38.38)			104 (36.49)	110 (38.60)		
Urban	120 (64.52)	61 (61.62)			181 (63.51)	175 (61.40)		
Degree of education			1.789	0.774			1.204	0.877
Primary school and below	58 (31.18)	34 (34.34)			92 (32.28)	89 (31.23)		
Junior high school	68 (36.56)	32 (32.32)			100 (35.09)	97 (34.04)		
High school or technical secondary school	26 (13.98)	17 (17.17)			43 (15.09)	39 (13.68)		
Junior college	17 (9.14)	10 (10.10)			27 (9.47)	32 (11.23)		
Bachelor degree or above	17 (9.14)	6 (6.06)			23 (8.07)	28 (9.82)		
Per capita monthly household income			2.334	0.506			1.067	0.785
<3,000 yuan	96 (51.61)	56 (56.57)			152 (53.33)	148 (51.93)		
≥3,000 yuan and <5,000 yuan	70 (37.63)	32 (32.32)			102 (35.79)	98 (34.39)		
≥5,000 yuan and <8,000 yuan	10 (5.38)	8 (8.08)			18 (6.32)	22 (7.72)		
≥8,000 yuan	10 (5.38)	3 (3.03)			13 (4.56)	17 (5.96)		
History of smoking			6.825	0.009			0.472	0.492
Yes	80 (43.01)	27 (27.27)			107 (37.54)	115 (40.35)		
No	106 (56.99)	72 (72.73)			178 (62.46)	170 (59.65)		
History of drinking			17.654	0.000			0.349	0.555
Yes	101 (54.30)	28 (28.28)			129 (45.26)	122 (42.81)		
No	85 (45.70)	71 (71.72)			156 (54.74)	163 (57.19)		
Concomitant hypertension			7.855	0.005			1.611	0.204
Yes	122 (65.59)	48 (48.48)			170 (59.65)	155 (54.39)		
No	64 (34.41)	51 (51.52)			115 (40.35)	130 (45.61)		
Concomitant dyslipidemia			9.018	0.003			1.580	0.209
Yes	108 (58.06)	39 (39.39)			147 (51.58)	132 (46.32)		
No	78 (41.94)	60 (60.61)			138 (48.42)	153 (53.68)		
Concomitant DKD			7.346	0.007			1.621	0.203
Yes	62 (33.33)	18 (18.18)			80 (28.07)	94 (32.98)		
No	124 (66.67)	81 (81.82)			205 (71.93)	191 (67.02)		
Concomitant DR			8.537	0.003			0.844	0.358
Yes	35 (18.82)	6 (6.06)			41 (14.39)	49 (17.19)		
No	151 (81.18)	93 (93.94)			244 (85.61)	236 (82.81)		
Medication regimen								
Biguanides	181 (97.31)	95 (95.96)	0.386	0.534	276 (96.84)	279 (97.89)	0.616	0.432
Dipeptidyl peptidase-4 inhibitors	94 (50.54)	47 (47.47)	0.242	0.622	141 (49.47)	146 (51.23)	0.175	0.675
Thiazolidinediones	87 (46.77)	42 (42.42)	0.493	0.482	129 (45.26)	122 (42.81)	0.349	0.555
Sulfonylureas	66 (35.48)	39 (39.39)	0.425	0.515	105 (36.84)	94 (32.98)	0.934	0.334
Alpha-glucosidase inhibitors	45 (24.19)	19 (19.19)	0.928	0.335	64 (22.46)	71 (24.91)	0.476	0.490
SGLT-2 receptor antagonists	39 (20.97)	25 (25.25)	0.681	0.409	64 (22.46)	73 (25.61)	0.778	0.378
Insulin and its analogues	21 (11.29)	14 (14.14)	0.488	0.485	35 (12.28)	44 (15.44)	1.190	0.275
Systolic blood pressure (mmHg)	141.07 ± 15.31	137.15 ± 14.23	2.108	0.036	139.71 ± 14.14	141.73 ± 14.22	1.701	0.090
Diastolic blood pressure (mmHg)	84.12 ± 8.52	83.41 ± 8.16	0.680	0.497	83.87 ± 8.42	84.09 ± 8.53	0.310	0.757
Fasting blood glucose (mmol/L)	9.43 ± 1.18	9.16 ± 1.15	1.855	0.065	9.34 ± 1.16	9.48 ± 1.09	1.485	0.138
Glycosylated hemoglobin (%)	8.74 ± 1.25	8.47 ± 1.14	1.789	0.075	8.65 ± 1.24	8.79 ± 1.21	1.364	0.173
Creatinine (μmol/L)	69.83 ± 20.14	65.08 ± 17.72	1.975	0.049	68.18 ± 16.08	67.29 ± 16.23	0.658	0.511
Total cholesterol (mmol/L)	5.44 ± 1.23	5.13 ± 1.28	1.997	0.047	5.33 ± 1.24	5.42 ± 1.25	0.863	0.389
Triglycerides (mmol/L)	1.73 ± 0.22	1.67 ± 0.19	2.296	0.022	1.71 ± 0.18	1.73 ± 0.17	1.364	0.173

### 3.3 Multivariable analysis based on training set data

Taking the occurrence of DPN in T2DM patients as the dependent variable (DPN = 1, non-DPN = 0), and factors such as age, body mass index, smoking history, drinking history, comorbidity of hypertension, comorbidity of dyslipidemia, comorbidity of DKD, comorbidity of DR, systolic blood pressure, creatinine, total cholesterol, and triglycerides as independent variables, collinearity was first excluded (exclusion criterion: variance inflation factor >5), followed by multivariate logistic regression analysis revealed that age (OR = 4.575, *p* = 0.028), body mass index ≥24.0 kg/m^2^ (OR = 2.463, *p* = 0.019), smoking history (OR = 2.585, *p* = 0.003), drinking history (OR = 2.717, *p* = 0.002), comorbidity of hypertension (OR = 2.925, *p* = 0.001), comorbidity of dyslipidemia (OR = 2.393, *p* = 0.014), comorbidity of DKD (OR = 2.602, *p* = 0.007), and comorbidity of DR (OR = 4.030, *p* = 0.010) were independent risk factors for DPN in T2DM patients (Table 2).

**Table 2:** Multivariable analysis based on training set data.

Factors	Description of assignment	*B*	S.E	Wald	*P*	OR	95% CI	
							Lower limit	Upper limit
Advanced age(1)	“no” = 0, “yes” = 1	1.521	0.692	4.823	0.028	4.575	1.178	17.775
Body mass index(1)	“<24.0 kg/m^2^” = 0, “≥24.0 kg/m^2^” = 1	0.902	0.384	5.521	0.019	2.463	1.161	5.225
History of smoking(1)	“no” = 0, “yes” = 1	0.950	0.325	8.548	0.003	2.585	1.368	4.886
History of drinking(1)	“no” = 0, “yes” = 1	1.000	0.320	9.739	0.002	2.717	1.450	5.090
Concomitant hypertension(1)	“no” = 0, “yes” = 1	1.073	0.317	11.485	0.001	2.925	1.572	5.441
Concomitant dyslipidemia(1)	“no” = 0, “yes” = 1	0.872	0.354	6.089	0.014	2.393	1.197	4.784
Concomitant DKD(1)	“no” = 0, “yes” = 1	0.956	0.353	7.345	0.007	2.602	1.303	5.195
Concomitant DR(1)	“no” = 0, “yes” = 1	1.394	0.537	6.725	0.010	4.030	1.405	11.554
Constant quantity		−2.273	0.451	25.389	0.000	0.103		

### 3.4 Construction of nomogram based on logistic regression results from training set

**Figure 2:**
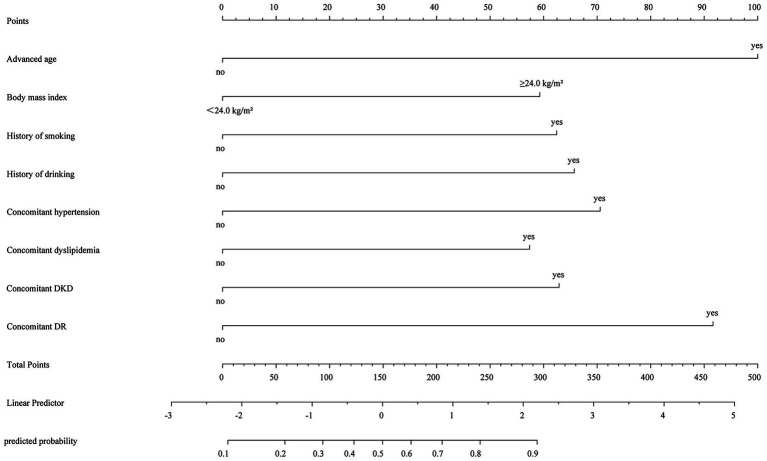
Construction of a nomogram based on the logistic regression analysis results of the training set.

A nomogram for predicting DPN in T2DM patients was constructed. Figure 2 shows the nomogram. Based on the factors of age, body mass index ≥24.0 kg/m^2^, smoking history, drinking history, comorbidity of hypertension, comorbidity of dyslipidemia, comorbidity of DKD, and comorbidity of DR, the corresponding points were determined from the “points” axis of the nomogram (Figure 2). By summing the individual scores for each factor (age, body mass index ≥24.0 kg/m^2^, smoking history, drinking history, comorbidity of hypertension, comorbidity of dyslipidemia, comorbidity of DKD, and comorbidity of DR), the total score is obtained, which can be used to calculate the individualized risk of DPN in T2DM patients.

### 3.5 Discrimination evaluation

**Figure 3:**
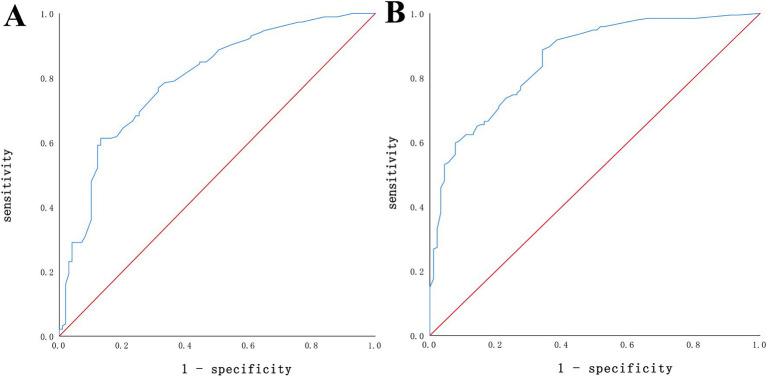
Differentiation evaluation. **(A)** ROC curve (training set); **(B)** ROC curve (validation set). The blue curve in the figure represents the ROC prediction curve of the model, and the red diagonal line represents the reference baseline (AUC = 0.5), indicating no predictive value. The farther the ROC curve is from the diagonal line, the stronger the discriminative ability of the model.

The area under the ROC curve for the training and validation sets was 0.795 (95% CI: 0.740–0.850) and 0.857 (95% CI: 0.812–0.903), respectively, as shown in Figure 3A (training set) and Figure 3B (validation set).

### 3.6 Consistency evaluation

**Figure 4:**
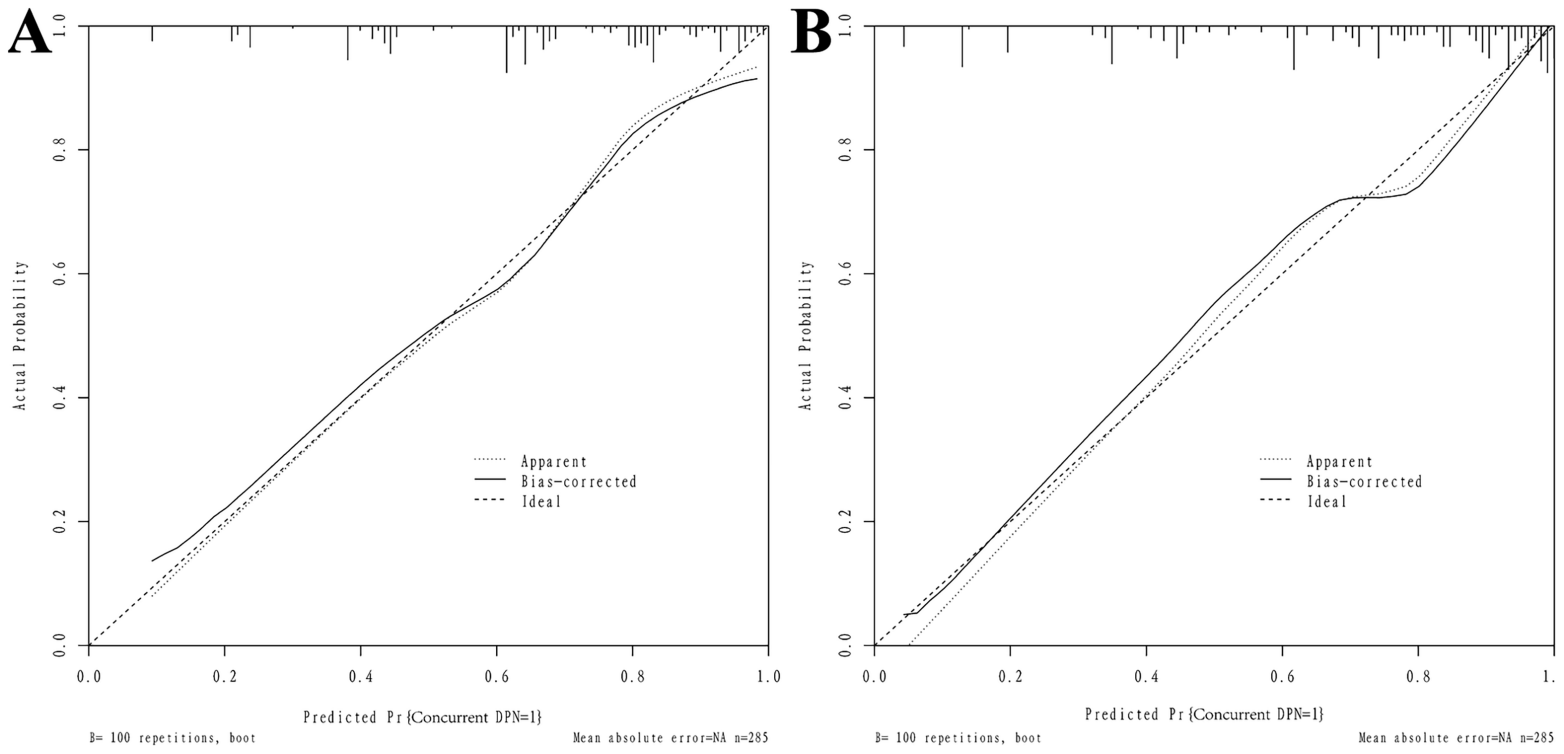
Consistency evaluation. **(A)** Calibration curve (training set); **(B)** Calibration curve (validation set). The Ideal line represents the ideal situation in which the predicted probability is completely consistent with the actual probability. The Apparent line represents the actual prediction curve of the model. The Bias-corrected line represents the curve after bias correction. The closer the Apparent line and Bias-corrected line are to the Ideal line, the better the consistency between the predicted probability and the actual observed probability of the model.

As shown in Figure 4A (training set) and Figure 4B (validation set), both the calibration curve and actual curve closely follow the ideal curve. The Hosmer-Lemeshow goodness-of-fit test for the training and validation sets was χ^2^ = 8.212, *p* = 0.413, and χ^2^ = 8.392, *p* = 0.396, respectively.

### 3.7 Clinical net benefit evaluation

**Figure 5:**
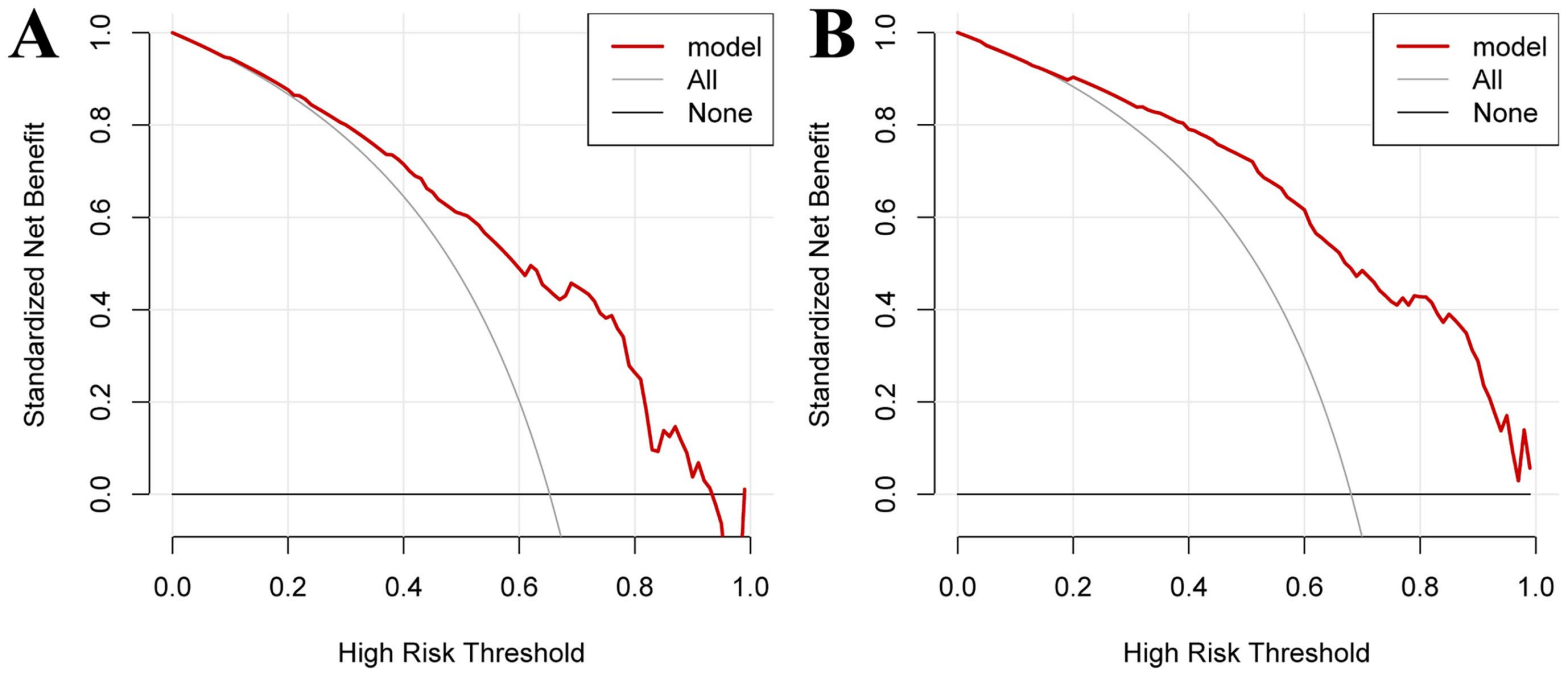
Clinical net benefit evaluation. **(A)** DCA curve (training set); **(B)** DCA curve (validation set). The Model curve represents the net benefit of the nomogram at different threshold probabilities. The All line indicates the assumption that all patients receive intervention, with the net benefit decreasing as the threshold probability increases. The None line indicates the assumption that no patients receive intervention, with the net benefit remaining at 0. The interval in which the Model curve is higher than the two baseline lines indicates that clinical intervention at this threshold probability can yield a positive net benefit. When the Model curve is above both the None line and the All line, it suggests that the use of this nomogram provides clinical net benefit.

For both the training and validation sets, when the threshold probability was between 0.22–0.96 and 0.18–1.00, respectively, the nomogram was more favorable for predicting the risk of DPN in T2DM patients. Figure 5A (training set) and Figure 5B (validation set).

## 4 Discussion

### 4.1 Purpose and significance of studying DPN in T2DM patients

Qi deficiency and blood stasis type DPN is essentially characterized by a root deficiency with an excess manifestation. Prolonged diabetes leads to the consumption of qi and damage to yin, resulting in phlegm and blood stasis obstructing the meridians. This blockage of the channels, combined with the internal generation of pathogenic toxins, gives rise to the development of the disease. DPN has been defined by the International Diabetes Foot Working Group as a major risk factor for diabetic foot (11). In this study, among the 285 T2DM patients in the training set, 186 (65.26%) had DPN, and 99 (34.74%) did not. Among the 285 T2DM patients in the validation set, 194 (68.07%) had DPN, and 91 (31.93%) did not. These results indicate that patients with T2DM have a high risk of DPN. This may be related to the continuous increase in the prevalence of T2DM in China; as the number of patients with T2DM increases, the risk of DPN also rises.

### 4.2 Independent risk factors for DPN in T2DM patients

(1) Advanced Age (OR = 4.575, *p* = 0.028): Research has shown that the incidence of DPN increases with age, with rates of 8.4, 22.7, 33.0, and 42.4% for patients aged 20–34, 35–49, 50–64, and ≥65 years, respectively, and the incidence is significantly correlated with age (12). Popescu et al. (13) also believed that age influences the occurrence of DPN. (2) Body Mass Index ≥24.0 kg/m^2^ (OR = 2.463, *p* = 0.019): A body mass index ≥24.0 kg/m^2^ indicates overweight or obesity, which is often associated with dyslipidemia. This can alter blood viscosity, lead to microvascular obstruction, and result in tissue ischemia and hypoxia, thereby increasing the risk of peripheral nerve damage and DPN. (3) Smoking History (OR = 2.585, *p* = 0.003): The harmful components such as nicotine in cigarette smoke can cause an inflammatory state, alter insulin sensitivity, and affect the function of pancreatic *β*-cells, which slows nerve conduction and leads to tissue ischemia and hypoxia, increasing the risk of DPN (14). The DPN risk is lower in non-smokers compared to mild, moderate, and heavy smokers (15). (4) Drinking History (OR = 2.717, *p* = 0.002): The damage to nerve cells caused by alcohol and its metabolites may explain the increased risk of DPN. (5) Comorbidity of Hypertension (OR = 2.925, *p* = 0.001): Hypertension can lead to small artery sclerosis, which may cause peripheral nerve damage. (6) Comorbidity of Dyslipidemia (OR = 2.393, *p* = 0.014): The free fatty acids in dyslipidemia can be associated with neurotoxic effects on neurons (16). Dyslipidemia can also affect atherosclerotic plaques, resulting in abnormal nerve perfusion and nerve function damage (17). Some scholars believe that scientific lipid-lowering treatments may help protect peripheral sensory nerves (18). (7) Comorbidity of DKD (OR = 2.602, *p* = 0.007): The pathophysiological mechanisms of DKD and DPN share similarities (19). (8) Comorbidity of DR (OR = 4.030, *p* = 0.010): DPN and DR share similar pathological mechanisms, particularly microcirculatory disorders (20).

### 4.3 Evaluation and validation of the nomogram

In this study, the area under the ROC curve for the training and validation sets was 0.795 (95% CI: 0.740–0.850) and 0.857 (95% CI: 0.812–0.903), respectively, indicating good discrimination of the nomogram. The calibration curve and actual curve for both the training and validation sets closely followed the ideal curve, demonstrating high consistency. The Hosmer-Lemeshow goodness-of-fit test for the training and validation sets yielded χ^2^ = 8.212 (*p* = 0.413) and χ^2^ = 8.392 (*p* = 0.396), respectively, indicating good fit of the nomogram. The threshold probabilities for both the training and validation sets ranged from 0.22–0.96 and 0.18–1.00, respectively, suggesting that the nomogram is more beneficial for predicting the risk of DPN in T2DM patients, enabling timely intervention and increased clinical benefit. The predictive factors in the nomogram, including advanced age, body mass index ≥24.0 kg/m^2^, smoking history, drinking history, comorbidity of hypertension, comorbidity of dyslipidemia, comorbidity of DKD, and comorbidity of DR, are all easily accessible by clinicians. Furthermore, this study was not a multicenter, large-sample investigation; all T2DM patients were from the same medical center, which may introduce selection bias. Future plans include collaborating with other centers to further improve and optimize the nomogram.

### 4.4 Comparison with previous DPN prediction models, innovation of this model, and applicable scenarios

In recent years, several studies have constructed prediction models for DPN. For example, Pan H et al. (21) constructed a nomogram for the individualized prediction of DPN based on multiple indicators, such as the duration of T2DM, glycated hemoglobin, and the insulin resistance index. Yu Q et al. (22) developed a nomogram for the individualized prediction of DPN by selecting multiple indicators, including diabetic retinopathy, diabetic foot, and diabetic nephropathy, based on a LASSO-logistic regression model. Compared with the above models, the present model has the following similarities and differences: (1) Similarities: all models use a nomogram to achieve individualized and quantitative prediction of the risk of DPN in patients with T2DM. (2) Differences: this model does not rely on traditional related variables such as the duration of T2DM and glycated hemoglobin, but instead incorporates more easily accessible clinical data, including smoking history, drinking history, hypertension, dyslipidemia, DKD, and DR, making it more suitable for promotion in primary medical institutions. In addition, in this study, the training set and validation set were strictly randomly divided at a ratio of 1:1, and the internal validation procedure was standardized. (3) Advantages: the areas under the ROC curve were 0.795 (95% CI: 0.740–0.850) and 0.857 (95% CI: 0.812–0.903), which were slightly higher than 0.789 (95% CI: 0.741–0.873) reported by Pan H et al. (21), and 0.780 (95% CI: 0.764–0.796) and 0.765 (95% CI: 0.739–0.791) reported by Yu Q et al. (22). These results indicate that the model achieved a good level of discrimination. Moreover, the predictive indicators are simple and easy to obtain, giving the model greater practical value.

The innovation of this study is mainly reflected in the fact that this model does not rely on traditional related variables such as the duration of T2DM and glycated hemoglobin, but instead includes more easily accessible clinical data, such as smoking history, drinking history, hypertension, dyslipidemia, DKD, and DR, which facilitates its application in primary medical institutions. Applicable scenarios: this model can be applied for DPN risk stratification among outpatients and inpatients with T2DM in endocrinology departments, and can provide a quantitative reference for clinicians to rapidly identify patients at high risk of DPN and formulate individualized intervention strategies.

### 4.5 Limitations

(1) In this study, DKD and DR were included as predictors of DPN in patients with T2DM. However, DPN, DKD, and DR are all parallel diabetic microvascular complications rather than temporally sequential exposure factors. Therefore, there is objectively a certain issue of circular reasoning, which may easily overestimate the apparent discriminative ability of the model. In addition, DKD and DR usually occur in patients with a longer duration of diabetes and advanced microvascular complications. As a result, this model may not be applicable to primary prevention of DPN or risk screening in early asymptomatic populations. Future studies should avoid using parallel microvascular complications as predictors and should focus more on factors such as duration of diabetes, glycemic control, and insulin resistance. (2) Key confounding variables were missing. As this study was a retrospective analysis of medical record data, we were unable to collect several recognized confounding variables associated with DPN, including duration of diabetes, glycated hemoglobin, insulin resistance index, homocysteine, and vitamin B12 levels. The absence of these confounding variables may have resulted in selection bias and, to some extent, affected the completeness, stability, and generalizability of the model. For example, duration of diabetes and glycated hemoglobin are relatively stable factors in the occurrence and progression of DPN, and their absence may reduce the model’s predictive ability for early DPN risk. In future studies, the above indicators should be supplemented and incorporated through prospective research to further validate and optimize the prediction model. (3) The data in this study were derived solely from the medical records of hospitalized patients at a single center, and the study adopted a retrospective design. Therefore, there may be a risk of information bias, such as incomplete medical record documentation, measurement errors in indicators, and bias in information collection. In addition, the single-center sample may limit the generalizability of the conclusions to other regions and medical institutions of different levels. Future multicenter prospective studies with standardized data collection procedures are recommended to further validate the robustness of this model.

In summary, advanced age, body mass index ≥24.0 kg/m^2^, smoking history, drinking history, comorbidity of hypertension, comorbidity of dyslipidemia, comorbidity of DKD, and comorbidity of DR are independent risk factors for DPN in T2DM patients. The nomogram based on these factors can effectively predict the risk of DPN in T2DM patients.

## Data Availability

The original contributions presented in the study are included in the article/supplementary material, further inquiries can be directed to the corresponding author.
